# Septal Thickness Does Not Impact Outcome After Hypertrophic Obstructive Cardiomyopathy Surgery (Septal Myectomy and Subvalvular Mitral Apparatus Remodeling): A 15-Years of Experience

**DOI:** 10.3389/fcvm.2022.853582

**Published:** 2022-06-15

**Authors:** Giuseppe M. Raffa, Eluisa La Franca, Carlo Lachina, Andrea Palmeri, Mariusz Kowalewski, Steven Lebowitz, Alessandro Ricasoli, Matteo Greco, Sergio Sciacca, Marco Turrisi, Marco Morsolini, Vincenzo Stringi, Gabriella Mattiucci, Michele Pilato

**Affiliations:** ^1^Department for the Treatment and Study of Cardiothoracic Diseases and Cardiothoracic Transplantation, IRCCS-ISMETT (Istituto Mediterraneo per i Trapianti e Terapie ad Alta Specializzazione), Palermo, Italy; ^2^Department of Cardiac Surgery, Central Clinical Hospital of the Ministry of Interior, Center of Postgraduate Medical Education, Warsaw, Poland; ^3^Innovative Medical Forum, Thoracic Research Center, Collegium Medicum, Nicolaus Copernicus University, Bydgoszcz, Poland; ^4^The University of Pittsburgh School of Medicine, Pittsburgh, PA, United States

**Keywords:** hypertrophic obstructive cardiomyopathy, mitral valve repair, mitral approach, systolic anterior motion, extensive myectomy

## Abstract

**Background:**

The aim of this study was to assess the impact of septal thickness on long-term outcomes of surgical treatment for hypertrophic obstructive cardiomyopathy (HOCM) and correction of mitral subvalvular anomalies.

**Methods:**

Sixty-six consecutive patients (58 ± 12 years, 56% female) undergoing extended septal myectomy and subvalvular mitral apparatus remodeling from 2007 to 2021 were retrospectively reviewed. Patients were divided into 2 groups according to septal thickness: moderate [< 18 mm, 29 patients (44%)] and severe [≥ 18 mm, 37 patients (56%)]. End points included survival, symptom improvement, reduction of left ventricle outflow tract (LVOT) gradient, resolution of mitral regurgitation (MR), and reoperation.

**Results:**

The mean interventricular septal thickness was 19 ± 3 mm, 15.8 ± 0.8 mm in patients with moderate and 21.4 ± 3.2 mm in those with severe hypertrophy. Preoperative data, intraoperative variables, postoperative complication rates, pre-discharge echocardiographic and clinical parameters did not differ between the two study groups [except for procedures involving the posterior mitral leaflet (*p* = 0.033) and septal thickness after myectomy (*p* = 0.0001)]. Subvalvular apparatus remodeling (secondary chordae of mitral valve resection and papillary muscle and muscularis trabecula procedures including resection, splitting, and elongation) was invariably added to septal myectomy (100%). Four (6%) procedures involved the posterior mitral leaflets. Mitral valve replacement was carried out in two patients (3%, *p* = 0.4). Reoperation for persistent MR was necessary in one patient (1%, *p* = 0.4). Neither iatrogenic ventricular septal defect nor in-hospital mortality occurred. During follow-up (mean 4.8 ± 3.8 years), two deaths occurred. NYHA class was reduced from 2.9 ± 0.7 to 1.6 ± 0.6 (*p* < 0.0001), the LVOT gradient from 89.7 ± 34.5 to 16.3 ± 8.8 mmHg (*p* < 0.0001), mitral valve regurgitation grade from 2.5 ± 1 to 1.2 ± 0.5 (*p* < 0.0001), and septal thickness from 18.9 ± 3.7 to 13.9 ± 2.7 mm (*p* < 0.0001).

**Conclusions:**

Regardless of septal thickness, subvalvular apparatus remodeling with concomitant septal myectomy can provide satisfactory long-term outcomes in terms of symptom improvement, LVOT obstruction relief, and MR resolution (without mitral valve replacement in most cases) in patients with HOCM.

## Introduction

Transaortic surgical septal myectomy (1) for the treatment of hypertrophic obstructive cardiomyopathy (HOCM) is associated with low operative morbidity and mortality, and reduction of the outflow gradient (2, 3). Systolic anterior motion (SAM) of the anterior mitral leaflet (AML) as a contributor to the pathophysiology of left ventricle outflow tract (LVOT) obstruction has been addressed (4–6) and in patients without severe hypertrophy (7, 8), SAM and mitral abnormalities (e.g., leaflet elongation and a wide array of malformations of the papillary muscles (PM) and chordae) may influence the dynamic obstruction of LVOT. In this context, the correction of subvalvular mitral apparatus abnormalities in addition to septal myectomy has been advocated (6, 8, 9) in order to abolish the LVOT gradient and to restore mitral competence. Although a matter of debate (10), these procedures include secondary chordae cutting (11), anterior leaflet plication (12) or extension (13), reorientation of PM (14), edge-to-edge repair (15), and mitral valve (MV) surgery (6, 7, 9, 16). MV replacement has been also reported (7, 16) if SAM and severe mitral regurgitation (MR) are challenging to manage.

The role of septal thickness in long-term outcomes of correction of subvalvular mitral apparatus abnormalities during myectomy is an under-studied matter of debate.

In this study we sought to assess the implications of septal thickness in long-term clinical and echocardiographic outcomes of patients requiring subvalvular MV remodeling (6, 9) in addition to extended septal myectomy.

## Materials and Methods

This retrospective and monocentric study was approved by the ethics committee of our institute (IRRB/35/16) and individual consent was obtained from each patient.

### Study Population

The study included 66 consecutive adult patients with HOCM treated surgically by extended septal myectomy and subvalvular MV remodeling between March 2007 and September 2021 at our institution. The patients had peak LVOT gradients ≥ 50 mmHg at rest or during stress and drug-refractory disabling symptoms (17) and were divided in 2 groups according to the septal thickness: moderate [< 18 mm, 29 patients (44%)] and severe [≥ 18 mm, 37 patients (56%)]. The cutoff of 18 mm was decided according to data available in the literature (7, 8). The 66 patients operated on represent 37% of 178 patients referred to our dedicated hypertrophic obstructive cardiomyopathy out-patient clinic who underwent surgery. Exclusion criteria were: history of mitral operation (including Mitraclip). Emergency operations and patients having undergone previous surgical myectomy or alcoholization were included.

Review of hospital records and analysis of preoperative history and reports, operative notes, and postoperative and follow-up echocardiography reports were carried out using a dedicated database.

### Diagnosis

All patients underwent preoperative Doppler transthoracic echocardiogram examination. Measurements of septal thickness were performed at end-diastole, in short-axis views from the mitral and mid left ventricle levels. Resting LVOT velocity was measured by continuous-wave Doppler of the outflow tract from an apical window. In HOCM patients who had a resting LVOT gradient < 30 mmHg but manifested drug refractory symptoms, provocative maneuvers such as the Valsalva were used during examination. Less symptomatic patients with LVOT gradient > 50 mmHg at rest underwent stress-echocardiogram. MV anatomy and function were evaluated by transthoracic and transesophageal echocardiogram (TEE) using an interactive approach. The degree of MR was measured by Doppler color flow imaging and quantitatively graded as mild (1 + /4 +), moderate (2 + /4 +), moderate-to-severe (3 + /4 +) and severe (4 + /4 +). A thorough analysis of the mitral subvalvular anomalies, including fibrotic and retracted secondary chordae inserted on the AML, abnormal chordae tendineae attached to the ventricular septum or free wall, and PM abnormalities was performed (6, 18). Cardiovascular magnetic resonance imaging providing detailed information on cardiac morphology, ventricular function, and myocardial tissue characteristics was not routinely performed. Most patients underwent pre-operative genetic and electrophysiology consults. Coronary anatomy was evaluated by angiograms and computed tomography scans. Intraoperative specimens were evaluated by pathologists.

### Surgical Technique

**FIGURE 1:**
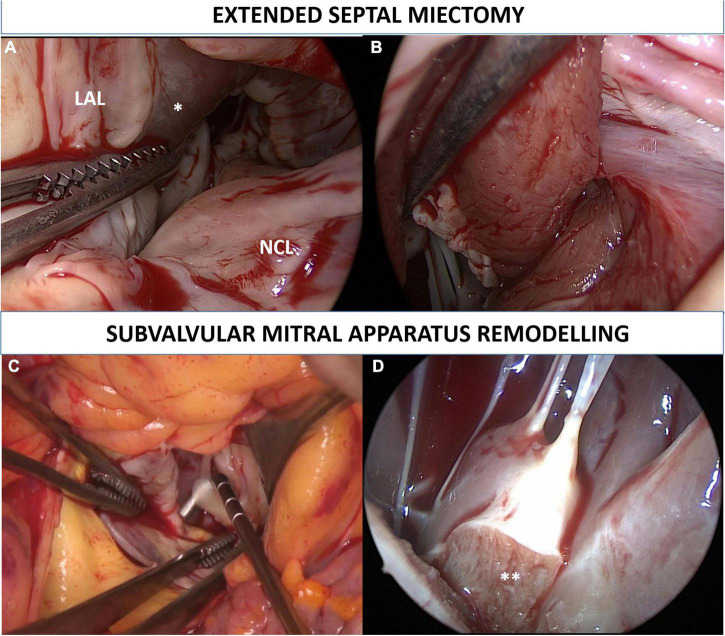
Extended septal myectomy. Surgical view of the left ventricle outflow tract before the procedure is depicted in panel **(A)**. Transaortic myectomy was performed in all patients starting at nadir of the right coronary sinus, and extended apically to achieve exposure of the papillary muscles bases. Muscular resection was extended toward the lateral ventricular wall up to the left trigone **(B)**. Subvalvular mitral apparatus remodeling included (1) resection of fibrotic, thickened, and agglutinated secondary chordae tendineae from the tip of the papillary muscles to the ventricular surface of the anterior mitral leaflet **(C)**; (2) resection of all anomalous muscular trabecula and splitting (**) of hypertrophied and thickened papillary muscles **(D)**. LAL, left aortic leaflet; NCL, non-coronary leaflet. *Interventricular septum bulging.

Complete surgical details have been reported previously (6), Figure 1. The operations were guided by TEE with particular attention paid to the septal anatomy and thickness, and MV apparatus. Briefly, all operations were performed through a full sternotomy, with aorta and right atrial cannulation and intermittent antegrade cold blood cardioplegia, and with mild hypothermia (34°C). Transaortic myectomy was performed in all patients starting at nadir of the right coronary sinus, and extended apically to achieve exposure of the PM bases. Muscular resection was extended toward the lateral ventricular wall up to the left trigone. The PM were carefully inspected to detect any hypertrophy, fusion, displacement, anomalies, or aberrances (e.g., bifurcation and fibrosis). Subvalvular mitral apparatus remodeling included (1) resection of all anomalous muscular trabecula and accessory PM; (2) resection of fibrotic, thickened, and agglutinated secondary chordae tendineae from the tip of the PM to the ventricular surface of the anterior mitral leaflet; and (3) splitting of hypertrophied and thickened PM. In cases of mitral leaflet repair, bicaval cannulation and a left or right atrial approach was used. The leaflet repair was done after myectomy and after the complete “release” of the AML obtained by the resection of diseased secondary chordae “anchoring” the leaflet itself to the PM and the septum. Further procedures on cardiopulmonary bypass were performed if necessary. After weaning from cardiopulmonary bypass and before protamine administration, a pharmacological stress test with adrenaline (from 5 to 10 mcg) was performed to assess for any residual LVOT gradient, SAM, and MR by the induction of tachycardia and systemic hypertension.

### Outcomes

Primary outcomes of the study were survival, symptom improvement [decrease in New York Heart Association (NYHA) functional class], reduction of LVOT gradient, and resolution of MR. Secondary outcomes included postoperative complications and reoperation for recurrence of LVOT obstruction and severe MR.

### Follow-Up Data

Yearly patient follow-up included physical examination, chest X-ray, electrocardiogram, transthoracic echocardiogram, and blood tests, and was performed in our dedicated outpatient clinic or by telephone interview. At September 1, 2021, the follow-up was 100% complete. The longest follow-up time was 14.6 years.

### Statistical Analysis

Continuous variables were expressed as mean, standard deviation, and range. Categorical variables were expressed as absolute value and percentage. The comparison between (1) patients with septal thickness < 18 mm and ≥ 18 mm; (2) NYHA, LVOT gradient, septum thickness, and residual MR before and after surgery were assessed by Chi-Square test, Fisher’s exact test, and logistic regression. Linear regression analysis was also applied to assess association with outcomes with septal thickness considered as a continuous predictor. Freedom from events at follow-up was assessed with Kaplan–Meier estimator and log-Rank test. Data management and analysis were done with SAS version 9.4. All *p*-values of < 0.05 were considered statistically significant.

## Results

### Patient Characteristics

The preoperative characteristics of the patients are depicted in Table 1. No major differences were observed between the two groups. The mean age was 58 ± 12 years, with 29 males and 37 females. Although patients were treated medically with β-blockers and/or calcium-channel blockers, all were symptomatic in NYHA class I-II (*n* = 15), III (*n* = 40), or IV (*n* = 11). Nine patients (13%) had a history of acute pulmonary edema and one (1%) was hospitalized at our institute due to cardiogenic shock. One patient underwent a second reoperation because of failure of previous surgeries performed elsewhere. History of previous alcoholization was recorded in one patient.

**TABLE 1:** Baseline characteristics.

	All, *n* = 66	Septal thickness < 18 mm (*n* = 29)	Septal thickness ≥ 18 mm (*n* = 37)	*P*-value
Female sex	37 (56)	20 (54)	17 (46)	0.08
Age (years)	58.4 ± 12.5 (26–80)	59.5 ± 11.7 (36–80)	57.5 ± 13.2 (26–75)	0.5
BMI (kg/m^2^)	27.6 ± 3.4 (20–39)	26.8 ± 3.1 (20–35)	28.3 ± 3.4 (21–39)	0.07
Family history for HOCM	22 (33)	9 (13)	13 (20)	0.7
Family history for SCD	23 (34)	9 (13)	14 (21)	0.6
Previous alcoholization	1 (1)	1 (1)	0 (0)	0.4
Syncope/lipothimia	15 (22)	6 (9)	9 (13)	0.7
Angina	33 (50)	12 (18)	21 (32)	0.3
Acute pulmonary edema	9 (13)	4 (6)	5 (7)	1
Pre-operative NYHA functional class	2.9 ± 0.7 (1–4)			0.6
I–II, n (%)	15 (22)	6 (9)	9 (13)	
III, n (%)	40 (61)	18 (28)	22 (33)	
IV, n (%)	11 (17)	5 (7)	6 (10)	
Pre-operative atrial fibrillation	37 (56)	13 (19)	24 (37)	0.1
Pre-operative SAM	53 (80)	22 (41)	31 (59)	0.5
Pre-operative ICD	13 (19)	3 (4)	10 (15)	0.1
Pre-operative LVEF (%)	64.2 ± 7.1 (40–83)	63.9 ± 5.3 (55–75)	64.5 ± 8.3 (40–83)	0.7
Pre-operative SPAP (mmHg)	27.4 ± 11.1 (19–70)	28.8 ± 12.8 (19–70)	26.4 ± 9.6 (20–58)	0.3
Pre-operative LVOT gradient* (mmHg)	89.7 ± 34.5 (28–174)	95.5 ± 35.6 (28–174)	85.1 ± 33.4 (40–165)	0.2
Pre-operative septal thickness (mm)	18.9 ± 3.7 (14–29)	15.8 ± 0.8 (14–17)	21.4 ± 3.2 (19–29)	0.7
Pre-operative MR grade	2.5 ± 1 (0–4)			0.4
1 + and 2 + /4 +	29 (43)	14 (21)	15 (22)	
3 + /4 +	25 (39)	9 (13)	16 (26)	
4 + /4 +	12 (18)	6 (9)	6 (9)	
Anterior mitral leaflet length (mm)	26.5 ± 3.8 (18–35)	26.2 ± 3.5 (21–32)	26.7 ± 4 (18–35)	0.6
Posterior mitral leaflet length (mm)	16.6 ± 3.7 (8–24)	17.5 ± 2.8 (11–22)	16 ± 4.2 (8–24)	0.1

*BMI, body mass index; HOCM, Hypertrophic obstructive cardiomyopathy; SCD, sudden cardiac death; NYHA, New York Heart Association; SAM, systolic anterior motion; ICD, implantable cardioverter defibrillator; LVEF, left ventricular ejection fraction; SPAP, systolic pulmonary artery pressure; LVOT, left ventricle outflow tract; MR, mitral regurgitation.*

*Peak gradient at rest.

*Continuous variables were expressed as mean, standard deviation and range.*

*Categorical variables were expressed as absolute value and percentage.*

The mean interventricular septal thickness was 19 ± 3 mm, 15.8 ± 0.8 mm in patients with moderate and 21.4 ± 3.2 mm in those with severe hypertrophy. Six (9%) patients had an extreme septal thickness (≥ 26 mm). Intraventricular peak gradient was 89 ± 34 mmHg. MR was graded 1 + and 2 + /4 + in 29 patients, 3 + /4 + in 25 patients, and 4 + /4 + in 12 patients. The average length of the AML and posterior mitral leaflet were 26 ± 3 mm and 16 ± 3 mm, respectively. A significant difference in neither preoperative clinical characteristics nor in echocardiographic data were detected between patients with moderate and severe septal thickness.

### Perioperative Outcomes

Surgical data and postoperative outcomes are summarized in Table 2. Intraoperative data, postoperative complication rate, and pre-discharge echocardiographic and clinical parameters did not differ between the two study groups [except for procedures involving the posterior mitral leaflet (*p* = 0.033) and septal thickness after myectomy (*p* = 0.0001)]. The analysis of the LVOT demonstrated thickened secondary chordae tendineae tractioning the AML to the interventricular septum in 64 patients (97%) and PM malformations in 55 patients (83%) including PMs hypertrophy (25 patients, 37%), accessory PMs (17 patients, 25%) and PMs fusion (13 patients, 20%). Surgical myectomy was performed in all patients (100%). Subvalvular mitral apparatus remodeling by means of secondary MV chordae resection (97%) and PM procedures (resection, splitting, and elongation; 83%) was added to myectomy where indicated. The mean number of resected secondary chordae was 3 ± 1. In 4 patients, due to redundancy and excessive tissue of the posterior mitral leaflet, resection and shortening of the leaflet itself was performed. Of note, all four of these patients were in the moderate septal thickness group. Intuitively, the septal thickness reduction was greater in the group with septal thickness less than 18 mm (12.7 ± 1.4 mm vs. 15 ± 2.9 mm, *p* = 0.0001). Two patients required valve replacement due to persistent severe MR after LVOT obstruction resolution. One patient, at the beginning of our experience in 2007, underwent early reoperation (during the same hospitalization) after a complicated postoperative course. A mechanical mitral prosthesis for recurrent SAM and severe MR was implanted on postoperative day 55 and the patient was discharged on post-operative day 6. In a 58-year-old female patient, a mechanical prosthesis was necessary after 3 failed attempts (including posterior leaflet shortening) to correct severe MR, although complete LVOT obstruction and SAM resolution were obtained. In this case, the AML was dysplastic and retracted. Aortic valve replacement (6 patients) and coronary artery bypass (2 patients) were also performed. No iatrogenic ventricular septal defect occurred. Intraoperative, in-hospital, and 30-day mortality were 0%. No patients developed complete atrioventricular block, but 3 patients (4%) required pacemaker implantation because of tachy-brady syndrome. Pre-discharge pharmacological treatment included b-blockers (56 patients, 85%), diuretics (52 patients, 79%), renin-angiotensin-aldosterone system inhibitors (23 patients, 35%) and calcium channel blockers (4 patients, 6%).

**TABLE 2:** Intraoperative results, early and long term outcomes.

	All, *n* = 66	Septal thickness < 18 mm (*n* = 29)	Septal thickness ≥ 18 mm (*n* = 37)	*P*-value
Aortic cross-clamp (min)	41.4 ± 10.6 (20–80)	41.9 ± 10.9 (20–65)	41.1 ± 10.6 (24–80)	0.7
Cardiopulmonary bypass (min)	56.5 ± 12.7 (25–105)	56.8 ± 13.7 (25–88)	56.2 ± 12.1 (38–105)	0.8
Resected cords (n)	3.5 ± 1.6 (0–8)	3.5 ± 1.6 (0–8)	3.5 ± 1.6 (0–7)	0.9
Procedures on papillary muscles (n)	1.3 ± 0.8 (0–3)	1.4 ± 0.9 (0–3)	1.2 ± 0.8 (0–2)	0.4
Mitral valve replacement	2 (3)	0	2 (3)	0.4
Intraoperative mitral valve replacement for MR	1 (1)	0	1 (1)	0.4
Posterior mitral leaflet shortening	4 (6)	4 (6)	0 (0)	**0.033**
Other procedures	6 (9)	4 (6)	2 (3)	0.3
Blood transfusion	37 (56)	17 (25)	20 (30)	0.8
Re-exploration	1 (1)	1 (1)	0 (0)	0.4
Low cardiac output syndrome	2 (3)	1 (1)	1 (1)	1
Sepsis	3 (4)	1 (1)	2 (3)	1
Post-operative atrial fibrillation	33 (50)	11 (16)	22 (34)	0.1
Post-operative PM implantation	3 (4)	2 (3)	1 (1)	0.5
Post-operative Complete AV block	0			
Iatrogenic ventricular septal defect	0			
Length of stay (days)	10.6 ± 8.3 (5–62)	10.1 ± 6 (5–33)	10.9 ± 9.8 (5–62)	1
Pre-discharge SAM	2 (3)	1 (1)	1 (1)	1
Pre-discharge				
LVOT gradient* (mmHg)	15.4 ± 8.5 (0–33)	14.7 ± 8 (0–30)	16 ± 8.8 (0–33)	0.5
Septal thickness (mm)	14 ± 2.6 (7–16)	12.7 ± 1.4 (10–16)	15 ± 2.9 (7–16)	**0.0001**
NYHA functional class	1.3 ± 0.5 (1–3)			0.7
I–II	63 (95)	27 (41)	36 (55)	0.7
III	3 (5)	2 (3)	1 (1)	
MR grade	1.2 ± 0.6 (0–3)			0.2
≤ 1 + /4 +	50 (75)	25 (37.5)	25 (37.5)	
2 + /4 +	15 (22)	4 (6)	11 (16)	
3 + /4 +	1 (3)	0	1 (3)	
Hospital mortality	0			
Follow-up				
SAM	7 (11)	2 (3)	5 (8)	0.5
LVOT gradient* (mmHg)	16.3 ± 8.8 (6–40)	18.2 ± 9.2 (6–40)	14.8 ± 8.2 (8–39)	0.1
Septal thickness (mm)	13.9 ± 2.7 (9–16)	12.9 ± 1.9 (10–18)	14.8 ± 3 (9–16)	**0.0068**
NYHA functional class	1.6 ± 0.6 (1–3)			0.9
I	27 (43)	11 (17)	16 (25)	
II	35 (54)	16 (25)	19 (29)	
III	2 (3)	1 (1)	1 (1)	
MR grade	1.2 ± 0.5 (0–4)			0.6
≤ 2 + /4 +	62 (98)	28 (44)	34 (54)	
4 + /4 +	1 (2)	0	1 (1)	
Reoperation at follow-up for MR	0			
Exitus at follow-up	2 (3)	1 (1.5)	1 (1.5)	1

*MR, mitral regurgitation; PM, pacemaker; AV, atrio-ventricular; SAM, systolic anterior motion. LVOT, left ventricle outflow tract; NYHA, New York Heart Association.*

*Peak gradient at rest.

*Continuous variables were expressed as mean, standard deviation and range.*

*Categorical variables were expressed as absolute value and percentage.*

*Bold values are referred to the significative p value.*

### Follow-Up Results

The mean follow-up time was 4.8 ± 3.8 years (range 0–14.5). Two deaths occurred, one at 83 days after surgery due to gastrointestinal bleeding and cardiogenic shock. The same patient was admitted in cardiogenic shock before HOCM surgery and a postmortem autopsy showed severe myocardial fibrosis, suggesting irreversible diastolic dysfunction. A late death occurred in one patient due to pneumonia 717 days after surgery. This patient underwent reoperation 97 days after HOCM surgery because of endocarditis of the aortic prosthesis and pacemaker lead. Follow-up data are available in Table 2. Recurrence of SAM was detected in 7 patients without significant obstruction. Severe MR occurred in one patient who is currently under close follow-up for asymptomatic MR. Two patients with obesity had NYHA III symptoms at follow-up.

**FIGURE 2:**
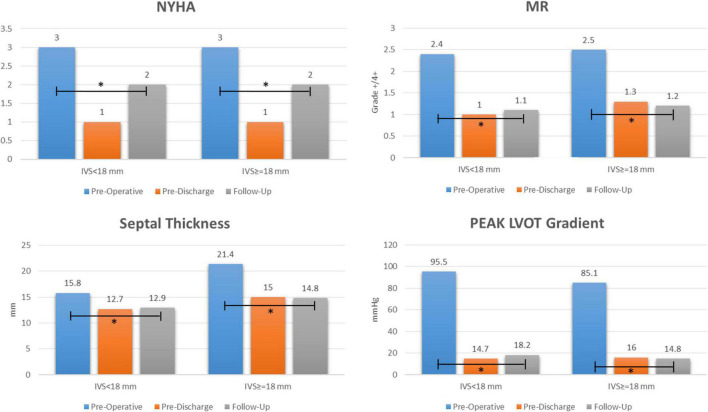
Early and long term outcome after extended septal myectomy and subvalvular mitral apparatus remodeling. NYHA: New York Heart Association; MR: mitral regurgitation; LVOT: left ventricle outflow tract; IVS: interventricular septum. “*” explains the events.

**FIGURE 3:**
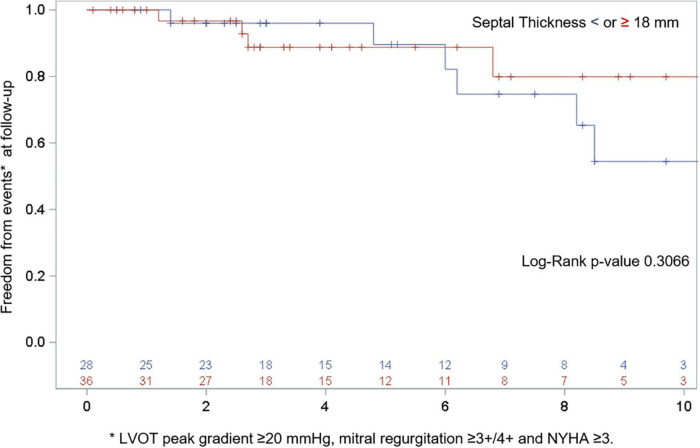
Freedom from composite end points at follow-up by means of recurrent LVOT obstruction (peak gradient ≥ 20 mmHg), MR ≥ 3 + /4 + and NYHA ≥ 3. *p = 0.0001.

At the last available follow-up, the survival and freedom from reoperation was 96%. NYHA class was reduced from 2.9 ± 0.7 to 1.6 ± 0.6 (*p* < 0.0001), the LVOT gradient from 89.7 ± 34.5 to 16.3 ± 8.8 mmHg (*p* < 0.0001), MV regurgitation grade from 2.5 ± 1 to 1.2 ± 0.5 (*p* < 0.0001), and septal thickness from 18.9 ± 3.7 to 13.9 ± 2.7 mm (*p* < 0.0001), Figure 2. Freedom from composite end points at follow-up by means of recurrent LVOT obstruction (peak gradient ≥ 20 mmHg), MR ≥ 3 + /4 + , and NYHA ≥ 3 are depicted in Figure 3. Seven patients (10%) showed a mean LVOT peak gradient of 35.8 ± 2.6 mmHg and a mean NYHA class 2.2 ± 0.4 (in two patients were detected a 2 + /4 + grade of MV regurgitation. All these patients are medically treated and strictly followed at out outpatients clinic. Details of these patients are depicted in Supplementary Table 1.

Further linear regression analysis of LVOT obstruction (*p* = 0.2232), SAM (*p* = 0.2982), and MR (*p* = 0.6468) recurrence with septal thickness as the continuous variable did not show any statistical relationship.

## Discussion

This study reports the long-term clinical and echocardiographic outcomes of subvalvular mitral anomalies correction with concomitant extended septal myectomy in HOCM patients with moderate (< 18 mm) and severe (≥ 18 mm) inteventricular septal thickness.

Our 15-year experience demonstrates that (1) HOCM is a wide spectrum of disease in which both septal hypertrophy and the MV apparatus invariably contribute to dynamic LVOT obstruction; (2) the subvalvular mitral apparatus (chordae and PM) promote SAM and contribute to obstruction; (3) regardless of septal thickness, subvalvular apparatus remodeling added to septal myectomy can provide satisfactory early- and long-term outcomes in terms of symptom improvement, LVOT obstruction, and MR resolution; (4) conservative procedures on the subvalvular mitral apparatus (“remodeling”) can correct SAM and MR and avoid, in most cases, MV replacement; (5) surgical remodeling of the subvalvular apparatus does not impair MV function at long-term follow-up; and (6) a dedicated HOCM team (dedicated clinical and interventional cardiologists, radiologists, surgeons, pathologists, and geneticists) is crucial to assess patients with hypertrophic cardiomyopathy and for planning the most appropriate treatment.

In our series, septal myectomy was performed in all cases, confirming the well-defined role of this technique for the relief of drug-refractory symptoms in patients with LV outflow obstruction (1–3, 17). Presently, the mortality rate after this procedure is below 1% (19, 20) and approaching zero in some series (21).

Concerns surround managing patients with moderate hypertrophy (7, 8, 11) in which the role of the MV in LVOT obstruction is predominant (4–6). Isolated myectomy should be considered cautiously because of iatrogenic ventricular septal defect occurrence and the insignificant relief of symptoms and LVOT obstruction (22). In fact, less marked septal basal hypertrophy may not be the sole cause of LVOT obstruction (8) and this clinical picture has been reported to be associated with mitral valvular and subvalvular anomalies (7, 8). These variants include changes in the MV leaflets (elongation, laxity, calcifications) and, more often, aberrancies of PM and secondary chordae (4, 5). We defined the complex anatomy of the LVOT in patients with HOCM as a “crowded” LVOT (18) in which, historically, replacement of the MV and subvalvular apparatus resection was the only viable option to treat the SAM-mediated obstruction and MR (22). In 2019, The Society of Thoracic Surgeons reported results of septal myectomy in the United States from a national database of more than 2,300 patients (16). About 1/3 of septal myectomy cases required MV operation (*n* = 801), including mitral repair (62%) and replacement (38%). Replacement compared to repair was associated with an increased risk of in-hospital mortality (4.4% vs. 1.9%) and MV surgery in addition to isolated septal myectomy (mortality 1.6%) was associated with an increased composite risk [OR 1.81, 95% confidence interval (CI): 1.39 to 2.36, *p* < 0.0001]. In the series by Lapenna et al. (7) the long-term mortality was higher in 23 patients with moderate septal thickness that required MV replacement plus septal myectomy compared to those with septal myectomy alone (*n* = 41) or septal myectomy plus MV repair (*n* = 12). In a prospective clinical trial (NCT02054221), 88 patients were randomized to MV replacement or repair during myectomy. At 2-year follow-up, the rates of overall survival, freedom from sudden cardiac death, and freedom from thromboembolic events were significantly higher in the repair group (23). Finally, the same group conducted a meta-analysis of over 2,762 patients with HOCM and MR (24) reporting a strong clinical benefit of MV repair compared to replacement in adult patients with HOCM.

The superiority of MV repair is well established for degenerative MV disease (25) and all efforts should be made to preserve the MV in patients with HOCM.

There is debate whether myectomy alone is enough to achieve the best outcome even in patients with moderate septal thickness. A dedicated high-volume center (10) reported on 1,486 surgical HOCM patients stratified by basal septal thickness (< 18 mm, *n* = 369; 18–21 mm, *n* = 612; and > 21 mm, *n* = 505). All patients underwent septal myectomy alone, regardless of septal thickness and degree of SAM-related MR. Concomitant MV procedures were performed for patients with intrinsic MV disease (66%) or insufficient intraoperative results in terms of residual mitral regurgitation (30%) and gradient (2%) after extended septal myectomy. In the group with septum < 18 mm, at early postoperative follow-up, moderate/severe mitral regurgitation was documented in only 2% of patients and SAM in 27.5%, with no difference compared to patients with a baseline septum ≥ 18 mm. The lack of long-term follow-up echocardiographic data is the major limitation of this study.

On the other hand, subvalvular mitral procedures are routinely performed at other dedicated HOCM centers with excellent results (6, 9, 11–13). For instance, the role of diseased secondary chordae in obstruction, especially in cases of a relatively thin septum, has been demonstrated by Ferrazzi et al. (11) Anomalous chordae resection (median of 3, range 1–8) associated with a shallow myectomy in 39 patients (with a ventricular septal thickness ≤ 19 mm) showed better clinical and hemodynamic results compared with a control group (only myectomy, 29 patients).

Our series provides long-term clinical and echocardiographic outcomes of procedures involving the subvalvular mitral apparatus during HOCM surgery. Release of the AML achieved by the technique described allows for coaptation of the mitral leaflet far from the septum and provides resolution of LVOT obstruction. Neither ventricular septal defect nor hospital mortality occurred. Intraoperative MV replacement because of a failed procedure due to severe MR recurrence was necessary in one patient. Pacemaker implantation rate was consistent with data from another single-center report (16). Our research highlights the complex anatomic interactions among the basal septal hypertrophy, mitral valvular and subvalvular abnormalities and SAM-in HOCM moreover when the septal thickness is less marked (91% of patients showed a septal thickness ≤ 24 mm), and contributes further data to the debate over surgical myectomy or alcoholization, though the use of the latter has increased in Europe in recent decades (25). This study also reports the definitive benefits of subvalvular mitral apparatus procedures added to septal myectomy in experienced centers. Preoperative planning, based on individual anatomic findings, and tailored surgical treatment resulted in excellent outcomes.

This is a single-center series of consecutive patients retrospectively reviewed. The small sample size and the lack of certain data (e.g., type of HOCM and hypertrophy localization, weight of the resected muscle, histopathologic analysis and cardiac magnetic resonance findings) should be considered its major limitations.

## Conclusion

Subvalvular apparatus remodeling added to septal myectomy can provide satisfactory early and long-term outcomes in terms of symptom improvement, LVOT obstruction relief, and MR resolution in patients with HOCM regardless of septal thickness.

## Data Availability Statement

The datasets presented in this article are not readily available due to the nature of this research, participants of this study did not agree for their data to be shared publicly, so supporting data is not available. Requests to access the datasets should be directed to GR, giuseppe.raffa78@gmail.com.

## Ethics Statement

The studies involving human participants were reviewed and approved by IRCCS-ISMETT. The patients/participants provided their written informed consent to participate in this study. Written informed consent was obtained from the individual(s) for the publication of any potentially identifiable images or data included in this article.

## Author Contributions

GR: conceptualization, formal analysis, methodology, supervision, validation, and writing—original draft. EF, CL, AP, AR, and MG: data curation and validation. MK and SL: methodology and writing—review and editing. SS, MT, VS, MM, and GM: data curation and supervision. MP: conceptualization, supervision, and validation. All authors contributed to the article and approved the submitted version.

## Conflict of Interest

The authors declare that the research was conducted in the absence of any commercial or financial relationships that could be construed as a potential conflict of interest.

## Publisher’s Note

All claims expressed in this article are solely those of the authors and do not necessarily represent those of their affiliated organizations, or those of the publisher, the editors and the reviewers. Any product that may be evaluated in this article, or claim that may be made by its manufacturer, is not guaranteed or endorsed by the publisher.
