# Supplementary material for: Septal Thickness Does Not Impact Outcome After Hypertrophic Obstructive Cardiomyopathy Surgery (Septal Myectomy and Subvalvular Mitral Apparatus Remodeling): A 15-Years of Experience
Source: Front Cardiovasc Med. 2022 Jun 15;9:853582. doi: 10.3389/fcvm.2022.853582 (PMC9242021; doi:10.3389/fcvm.2022.853582)
Supplement: Supplementary file 1 [file Table_1.DOCX]

**Supplemental table 1.** Baseline and follow-up data in patients with left ventricle outflow tract obstruction (residual peak gradient ≥ 30 mmHg and ≤40 mmhg) after surgical myectomy and mitral apparatus remodeling

| **Age at operation** | **Sex** | **BMI** | **NYHA**  **pre** | **NYHA**  **post** | **NYHA**  **FUP** | **Peak**  **Gradient**  **Pre-OP**  **(mmHg)** | **Peak**  **Gradient**  **Post-OP**  **(mmHg)** | **Peak**  **Gradient**  **FUP**  **(mmHg)** | **IVS**  **Pre-OP**  **(mm)** | **IVS**  **Post-OP**  **(mm)** | **IVS**  **FUP**  **(mm)** | **MR grade**  **Pre-OP** | **MR grade**  **Post-OP** | **MR grade**  **FUP** | **FUP time**  **(years)** | **Current pharmacological**  **treatment** | **Note** |
| --- | --- | --- | --- | --- | --- | --- | --- | --- | --- | --- | --- | --- | --- | --- | --- | --- | --- |
| 45 | F | 27.4 | 3 | 1 | 2 | 174 | 18 | 33 | 16 | 14 | 12 | 1+/4+ | 1+/4+ | 1+/4+ | 8 | metoprolol | 3^rd^ reoperation |
| 45 | F | 35.5 | 3 | 3 | 3 | 106 | 27 | 40 | 14 | 14 | 15 | 1+/4+ | 1+/4+ | 1+/4+ | 8 | Bisoprolol, furosemide | Persistence of severe obesity |
| 68 | F | 26.8 | 3 | 2 | 2-3 | 47 | 18 | 39 | 19 | 13 | 16 | 3+/4+ | 1+/4+ | 2+/4+ | 6 | Bisoprolol, furosemide, spironolactone, warfarin | Episodes of atrial arrhythmias* |
| 54 | M | 27.8 | 2 | 1 | 2 | 80 | 0 | 35 | 16 | 14 | 15 | 1+/4+ | 0+/4+ | 1+/4+ | 5 | metoprolol |  |
| 63 | F | 26 | 2 | 1 | 2 | 142 | 15 | 36 | 16 | 13 | 12 | 3+/4+ | 2+/4+ | 2+/4+ | 4 | Metoprolol, furosemide, spironolactone |  |
| 73 | F | 24.9 | 2 | 1 | 2 | 66 | 14 | 34 | 20 | 16 | 13 | 3+/4+ | 2+/4+ | 1+/4+ | 2 | Enalapril, bisoprolol, furosemide |  |
| 64 | F | 23.4 | 4 | 1 | 2 | 73 | 26 | 34 | 27 | 17 | 17 | 4+/4+ | 2+/4+ | 1+/4+ | 1 | Metoprolol, furosemide |  |

BMI=body mass index; NYAH=New York Heart Association; FUP=follow-up; Pre-Op=pre-operative; Post-OP=post-operative; IVS=inter ventricular septum; MR= mitral regurgitation. * current evaluation for ablation.
